# LIVING WITH YOUNG ONSET DEMENTIA IN RESIDENTIAL CARE: ANALYSIS OF DATA FROM THE SWEDISH DEMENTIA REGISTER

**DOI:** 10.1093/geroni/igae098.4015

**Published:** 2024-12-31

**Authors:** Linda Johansson, Therése Bielsten, Jimmy Lindberg, Deborah Finkel

**Affiliations:** School of Health and Welfare, Jönköping University, Jönköping, Jonkopings Lan, Sweden; Jönköping University, Jönköping, Jonkopings Lan, Sweden; Jönköping University, Jönköping, Jonkopings Lan, Sweden; University of Southern California, Los Angeles, California, United States

## Abstract

Approximately 10,000 people in Sweden have Young Onset Dementia (YOD), i.e., dementia onset before the age of 65. As a result of disease progression, relocation to a residential care facility might be necessary. In Sweden, age-adapted residential facilities are only available in larger cities, and the specific needs of persons living with YOD might not be adequately addressed. The study aimed to describe characteristics of residents with YOD living in residential care and the quality of care. National quality registry data from the Swedish Dementia Register (SveDem) between 2012 and 2022 were used. In total, 124 people with YOD living in residential care units designed for persons with dementia (n=109) or in residential care facilities for older persons (n=15) were identified. The average age of admittance was 61.3±3.9 years, with no significant differences found between women and men. On average, six medications were used, and a medication review had been conducted within the past 12 months for 84% of the participants. Care received in accordance with one’s life story (71%) and individual environmental adaptations (64%) were common. However, few participants (22%) had advanced care plans, and restraints were present in 44% of the persons with YOD. While it is not possible to draw general conclusions from this small study, it indicates that person-centered care is central in residential care for persons with YOD in Sweden. Future research should focus on increasing the understanding of why staff use restraints to such a high extent and how this practice can be prevented.

